# miR-155, a Modulator of FOXO3a Protein Expression, Is Underexpressed and Cannot Be Upregulated by Stimulation of HOZOT, a Line of Multifunctional Treg

**DOI:** 10.1371/journal.pone.0016841

**Published:** 2011-02-03

**Authors:** Mayuko Yamamoto, Eisaku Kondo, Makoto Takeuchi, Akira Harashima, Takeshi Otani, Kazue Tsuji-Takayama, Fumiyuki Yamasaki, Hiromi Kumon, Masayoshi Kibata, Shuji Nakamura

**Affiliations:** 1 Cell Biology Institute, Research Center, Hayashibara Biochemical Laboratories Inc., Okayama, Japan; 2 Department of Pathology, Okayama University Graduate School of Medicine, Dentistry and Pharmaceutical Sciences, Okayama, Japan; 3 Division of Oncological Pathology, Aichi Cancer Center Research Institute, Nagoya, Japan; 4 Kurashiki Medical Center, Kurashiki, Japan; 5 Department of Urology, Okayama University Graduate School of Medicine, Dentistry and Pharmaceutical Sciences, Okayama, Japan; University of Colorado Denver, United States of America

## Abstract

MicroRNAs (miRNAs) play important roles in regulating post-transcriptional gene repression in a variety of immunological processes. In particular, much attention has been focused on their roles in regulatory T (Treg) cells which are crucial for maintaining peripheral tolerance and controlling T cell responses. Recently, we established a novel type of human Treg cell line, termed HOZOT, multifunctional cells exhibiting a CD4^+^CD8^+^ phenotype. In this study, we performed miRNA profiling to identify signature miRNAs of HOZOT, and therein identified miR-155. Although miR-155 has also been characterized as a signature miRNA for FOXP3^+^ natural Treg (nTreg) cells, it was expressed quite differently in HOZOT cells. Under both stimulatory and non-stimulatory conditions, miR-155 expression remained at low levels in HOZOT, while its expression in nTreg and conventional T cells remarkably increased after stimulation. We next searched candidate target genes of miR-155 through bioinformatics, and identified *FOXO3a*, a negative regulator of Akt signaling, as a miR-155 target gene. Further studies by gain- and loss-of-function experiments supported a role for miR-155 in the regulation of FOXO3a protein expression in conventional T and HOZOT cells.

## Introduction

MicroRNA (miRNA) comprises a specialized subset of small cytoplasmic noncoding RNAs between 19 and 24 nucleotides in length. miRNAs have been found in animals, plants, and viruses, with over 700 identified in humans [1]. miRNA genes are first transcribed as pri-miRNAs, and then processed to shorter hairpin-shaped pre-miRNAs (∼70–90 nt) by RNase Drosha, and finally cleaved into mature single-stranded miRNAs (∼22 nt) by RNase Dicer. The processed miRNAs most frequently bind to regulatory sequences in the 3′- untranslated region (UTR) of target mRNAs through imperfect base-pairing, resulting in translational inhibition or degradation of protein-coding transcripts [2]. Bioinformatics and cloning studies have estimated that miRNAs may regulate 30% of all human genes [3] [4]. Thus, miRNAs constitute a regulatory network which acts as a binary off-switch or a rheostat to regulate post-transcriptional gene repression in a variety of processes [5] [6]. For example, miRNAs are critically important in the immune system, controlling the development, differentiation, apoptosis, and effector functions of immune cells as well as their involvement in tumor pathogenesis [7] [8].

The roles of miRNA in immunology have been studied in Treg cells [9]. The first indication of miRNA involvement in Treg differentiation came from findings in Dicer-deficient mice, which exhibited reduced numbers of Treg cells and immune pathology such as colitis, and lung and liver inflammation [10]. Further experiments using Treg-specific deletion of Dicer or Drosha demonstrated that these knockout mice succumbed to autoimmunity in a manner indistinguishable from Foxp3-deficient mice, indicating a requirement for functional miRNA machinery in Treg homeostasis as well as in suppressive function [11] [12] [13]. Treg cells express a characteristic set of miRNAs distinct from that of naïve CD4 T cells but which overlap with that of activated T cells [10]. Among them, specific miRNA molecules have been analyzed as responsible for Treg cell biology. For example, miR-155, which is overexpressed in Treg cells, plays an important role in Treg homeostasis and overall survival through directly targeting a negative regulator for IL-2 signaling, SOCS1 [14]. Also, miR-142-3p, which is underexpressed in Treg cells, plays an antagonizing role in the suppressor function of Treg through regulation of cyclic AMP [15].

A multifunctional Treg cell line, termed HOZOT, was generated by co-culturing human umbilical cord blood cells with mouse stromal cell lines in the absence of exogenous IL-2 or other cytokines [16]. HOZOT were characterized by a CD4/CD8 double positive phenotype, a unique property compared with other T-cell subsets. Furthermore, HOZOT possessed suppressor/helper/cytotoxic activities, anergic properties, and produced high levels of IL-10 [17] [18] [19], [20]. Previously, we performed mRNA profiling to identify signature cytokines of HOZOT. We found that HOZOT's expression pattern was multifaceted, resembling Th1 and Th2 T cells, CD8^+^ CTL and NK cells, and Tregs. In particular, two chemokines, IL-8 and RANTES, were produced at high levels by HOZOT [21]. Through this profiling study, however, we could not identify specific molecules responsible for controlling HOZOT's differentiation or functions.

In this study, we further characterized HOZOT by miRNA profiling and found a unique expression pattern of miR-155. We propose that HOZOT maintains a high level of expression of FOXO3a protein by down-modulating miR-155 expression.

## Results

### MicroRNA profiling for HOZOT-17

**Figure 1:**
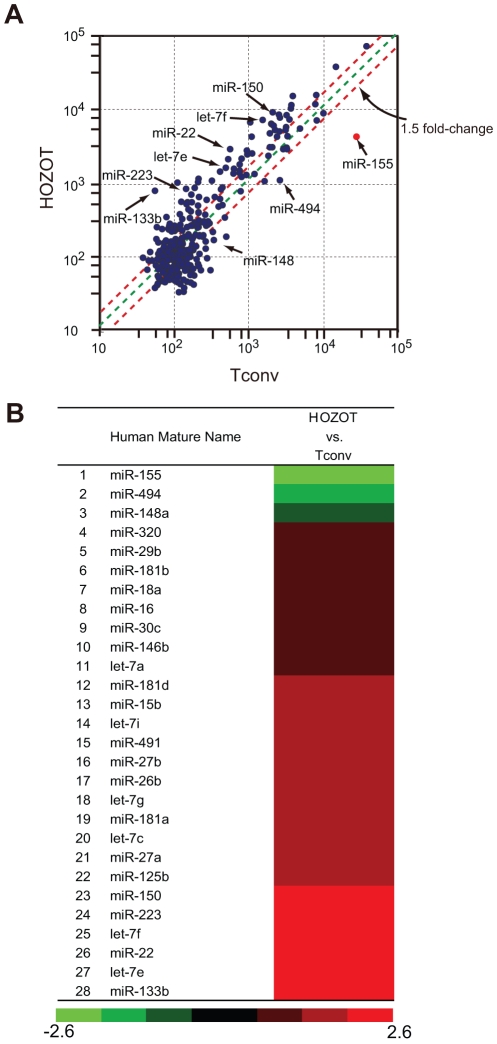
Downregulated expression of miR-155 in HOZOT revealed by miRNA microarray analysis. *A*. miRNA array comparison of HOZOT versus Tconv cells. HOZOT-17 was used as a representative HOZOT cell line and an antibody-activated T cell line (prepared from the same UCB source) was used as Tconv cells. The expression value of each human miRNA was plotted as a scatter plot, and threshold lines were drawn at 1.5-fold expression change. *B*. miRNA profiling results displayed as a heatmap. Upregulated and downregulated miRNAs in HOZOT-17 in comparison with Tconv are shown in green and red, respectively.

We used microarray analysis to compare miRNA expression profiles of HOZOT with those of activated T cells, so called conventional CD4^+^ T (Tconv) cells (Figure 1A). HOZOT-17, a representative HOZOT cell line, was used for profiling. Tconv cells were prepared from the same UCB source as HOZOT-17. CD4^+^ cells were activated with anti-CD3 antibody plus anti-CD28 antibody (CD3/CD28) and cultured for at least seven days in the presence of IL-2. The chip used for this analysis was a mirVanaTM miRNA Bioarray system containing a total of 328 probes for human miRNAs. The results are shown in Figure 1A. In HOZOT-17, 73 miRNAs were expressed at levels more than 1.5-fold higher than in Tconv cells, whereas only three miRNAs (miR-155, miR-494, and miR-148a) were expressed at levels lower than in Tconv cells. Twenty-eight miRNAs which changed significantly between the two samples are displayed as a heat map in Figure 1B. They are as follows: let-7a, let-7c, let-7e, let-7f, let-7g, let-7i, miR-15b, miR-16, miR-18a, miR-22, miR-26b, miR-27a, miR-27b, miR-29b, miR-30c, miR-125b, miR-133b, miR-146b, miR-148a, miR-150, miR-155, miR-181a, miR-181b, miR-181d, miR-223, miR-320, miR-491, and miR-494.

Among the up-regulated miRNAs, let-7 family members (let-7a, c, e, f, and i) are tumor suppressor genes and are underexpressed in Treg and activated T cells [10]. miR-150 was shown to be downregulated in activated T cells as well as in Treg cells. Therefore, these miRNAs have expression patterns which are the opposite of those seen in HOZOT. miR-146 and miR-223 are upregulated genes in HOZOT, Treg and activated T cells. Among down-regulated genes, miR-155 was markedly underexpressed in HOZOT. Expression of miR-155 was 6.3-fold lower in HOZOT than in Tconv cells. miR-155 is one of the best-characterized miRNAs in hematopoietic cells and its expression in T cells, B cells, and myeloid cells is greatly enhanced by activation [7]. HOZOT's unusual expression pattern of miR-155 was intriguing and we further explored the nature of miR-155 expression in HOZOT.

### miR-155 expression is non-responsive to TCR stimulation and correlates with FOXP3 expression

**Figure 2:**
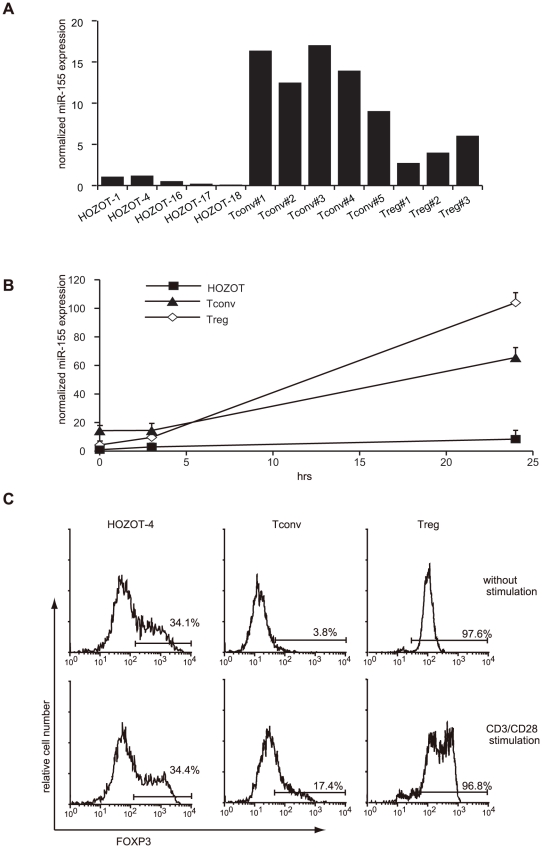
Underexpression and non-responsiveness of miR-155 are unique features of HOZOT and correlated with FOXP3 expression. *A*. Decreased expression of miR-155 in HOZOT was validated by real-time PCR using HOZOT cell lines (n = 5) in comparison with Tconv cell lines (n = 5) and Treg cell lines (n = 3). All RNA samples were prepared from the cell lines before re-stimulation. The ratio of miR-155/U6 small nuclear RNA (snRNA) in HOZOT-1 was defined as 1.0. *B*. miR-155 expression does not respond to stimulation in HOZOT. HOZOT (n = 5), Tconv (n = 3), and Treg (n = 3) cells were re-stimulated with CD3/CD28. miR-155 expression was monitored at zero, three and 24 hrs after re-stimulation. The ratio of miR-155/U6 snRNA in HOZOT was defined as 1.0. *C*. Expression of FOXP3 in HOZOT is not responsive to stimulation. Flow cytometric analysis of FOXP3 expression was assayed by intracellular staining 24 hrs after re-stimulation. Values in histograms indicate the percentage of FOXP3^+^ cells.

To validate the results of the miRNA array, we semi-quantitatively measured miR-155 expression by qRT-PCR. In addition to HOZOT-17, additional T cell lines, other HOZOT cells, Tconv cells, and Treg cells were included in this analysis. Comparisons of miR-155 expression among these T cell lines revealed that under resting conditions, Tconv cells expressed miR-155 at the highest levels, Treg cells at the second, and HOZOT at the lowest (Figure 2A). Since the most prominent feature of miR-155 is its high responsiveness to TCR stimulation, the kinetics of miR-155 expression were monitored after TCR re-stimulation among three types of T cell lines. As shown in Figure 2B, miR-155 expression in HOZOT was only marginally elevated, whereas sharp increases were observed in both Tconv and Treg cells. As one of the mechanisms controlling miR-155 expression, Foxp3 is thought to act as a transcriptional activator through its binding to an intronic element of *BIC*, a gene encoding the precursor transcript of miR-155 [22] [23]. The forced expression of Foxp3 results in miR-155 up-regulation [14]. Therefore, we examined whether FOXP3 expression correlated with miR-155 expression in HOZOT. As previously reported, FOXP3 expression was detected in HOZOT to some extent and its expression levels varied from one HOZOT line to another. As shown in Figure 2C, FOXP3 protein expression was markedly enhanced in Treg and moderately upregulated in Tconv cells by CD3/CD28 stimulation, but almost no increase in FOXP3 expression was observed in HOZOT. Therefore, in HOZOT, non-responsiveness to TCR stimulation was a feature shared by miR-155 and FOXP3.

### Reporter assays for miR-155 target genes selected by data mining

**Figure 3:**
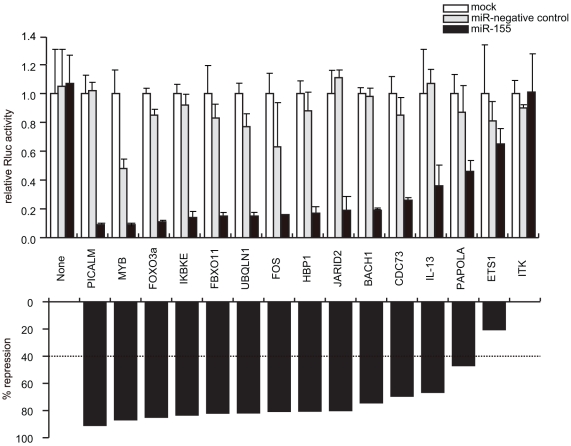
Repression of specific target genes by miR-155 through direct 3′- UTR interactions. *A* luciferase reporter assay was performed by transiently transfecting JURKAT cells with plasmids containing the 3′-UTR sequence of each target gene which contained at least one miR-155 binding site. A negative control plasmid (none) contained no 3′-UTR. Cells were co-transfected further with the precursor of miR-155 or miR-negative control, or without them (mock). *Renilla* luciferase activities were normalized to firefly luciferase. All data are from triplicate sets representing at least three independent experiments.

To further explore the relevance of underrepresented expression of miR-155, *in silico* analysis was performed to predict miR-155 targets. We combined two approaches, mRNA expression profiling, and miR-155 target prediction web program to select candidate genes. These approaches were based on the criteria that mRNAs of such target genes should be expressed at relatively high levels and contain miR-155 binding sites in their 3′-UTR. As a first screening, 2000 genes were selected by target prediction program and then 100 genes by mRNA microarray profiling [21]. Among 100 genes, we focused on 19 genes whose functions have been associated with T-cell biology or hematopoiesis and whose 3′-UTR sequences contain tandem predicted binding sites of miR-155. They include *BACH1, CDC73, ETS1, FBXO11, FOS, FOXO3a, HBP1, IKBKE, IL-13, IRF2BP2, ITK, JARID2, MYB, PAPOLA, PICALM, RANTES, RUNX1, RUNX2*, and *UBQLN1* (Table 1 and data not shown). Using reporter genes, we tested whether their expression was modulated by miR-155. Each reporter construct was introduced into JURKAT, whose miR-155 expression was barely detectable, and the effects of miR-155 or negative control miRNA were examined (Figure 3). The results showed that the relative luciferase activities of 13 out of 19 reporters were decreased by 40 to 95% following introduction of miR-155. In contrast, the miRNA negative control had almost no significant effect on luciferase activity except in the case of a *MYB* gene construct (52% suppression). A negative control construct (none), which had no 3′-UTR, showed no suppression even after introduction with miR-155.

**Table 1:** Predicted targets of miR-155.

Target gene	Gene ID	Gene name	Conserved sites	Poorly conserved sites
BACH1	NM_001186	BTB and CNC homology 1, basic leucine zipper transcription factor 1	3	1
CDC73	NM_024529	cell division cycle 73, Paf1/RNA polymerase II complex component, homolog (*S. cerevisiae*)	0	1
FBXO11	AL117620	F-box protein 11	1	1
FOS	BC004490	v-fos FBJ murine osteosarcoma viral oncogene homolog	1	0
FOXO3a	N25732	forkhead box O3	0	4
HBP1	AF019214	HMG-box transcription factor 1	1	0
IKBKE	NM_014002	inhibitor of kappa light polypeptide gene enhancer in B-cells, kinase epsilon (IKKε)	2	0
IL13	NM_002188	Interleukin 13	0	1
JARID2	BG029530	jumonji, AT rich interactive domain 2	2	0
MYB	NM_005375	v-myb myeloblastosis viral oncogene homolog (avian)	2	0
PAPOLA	NM_032632	poly(A) polymerase α	0	2
PICALM	AL135735	phosphatidylinositol binding clathrin assembly protein	0	3
UBQLN1	NM_013438	ubiquilin 1	0	2

Conserved sites: number of broadly conserved sites among vertebrates.

Poorly conserved sites: number of human target sites.

### FOXO3a is a direct target of miR-155

**Figure 4:**
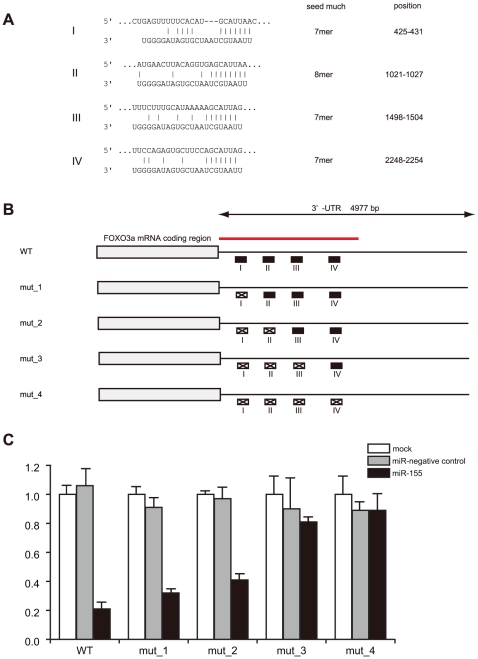
FOXO3a mRNA contains the four binding sites in the 3′- UTR relevant for miR-155 mediated repression. *A*. Four predicted interaction sites (I through IV) of miR-155 were found within the 3′-UTR of *FOXO3a* mRNA. Putative pairings between target genes (top sequence) and hsa-miR-155 (bottom sequence) are shown as a 7-mer or 8-mer seed match. *B*. Reporter constructs with or without mutation in the 3′UTR of FOXO3a mRNA are shown by indicating the mutated sites of each construct marked with an X. The part of the 3′-UTR used for cloning is depicted with a red line. *C*. A luciferase reporter assay was performed by transiently transfecting JURKAT cells with plasmid constructs with (mut_1 through mut_4) or without mutation (WT) and then by co-transfecting them with the precursor of miR-155 or miR-negative control as described in Figure 3. *Renilla* luciferase activities were normalized to firefly luciferase. All data are from triplicate sets representing at least three independent experiments.

Next, we focused on one of the target genes, *FOXO3a*, which is another member of the forkhead family of transcription factors and distinct from *FOXP3*, because its 3′-UTR contains four predicted binding sites (Figure 4A). Although not conserved between human and mouse, these sites are conserved among higher mammalian animal species as follows: Region I is conserved among human, chimpanzee, rhesus, and guinea pig. Region II is conserved among human, chimpanzee, and rhesus. Region III is most conserved in many species, such as human, chimpanzee, rhesus, rabbit, shrew, hedgehog, and cow. Region IV is conserved only in human and chimpanzee. To demonstrate a direct interaction between miR-155 and the FOXO3a 3′-UTR, we sequentially mutated these four binding sites corresponding to the miR-155 seed regions (Figure 4B). Without any mutation, miR-155 repressed 80% of luciferase activity of the reporter construct. As the mutation sites increased, the repression by miR-155 was gradually cancelled from 64% (mut_1) through 10% (mut_3) and eventually to no repression (mut_4). These results suggest that miR-155 could repress human FOXO3a expression through the direct interaction with these four conserved sites.

### FOXO3a protein was expressed at high levels in HOZOT

**Figure 5:**
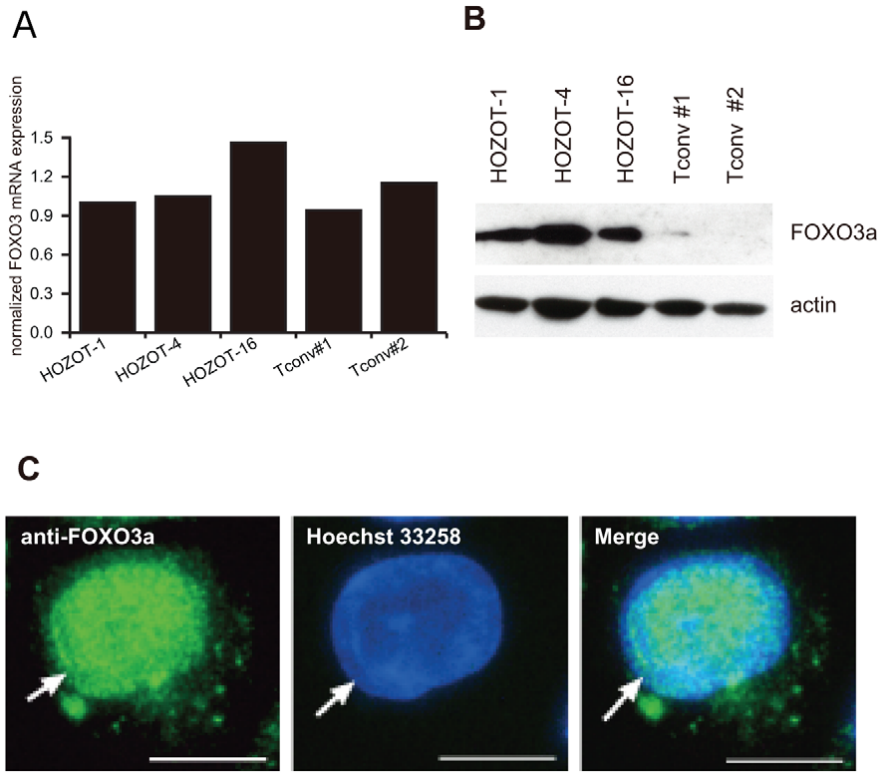
FOXO3a protein was overexpressed in HOZOT. *A, B.* Expression of FOXO3a mRNA (A) or protein (B) was measured in HOZOT and Tconv cells by qRT-PCR or Western blotting, respectively. Three HOZOT cell lines and two Tconv cell lines were compared. Ratio of mRNA expression of FOXO3a/18S rRNA in HOZOT-1 was defined as 1.0. *C*. FOXO3a protein was detected immunohistochemically in HOZOT cells. Cytospins of HOZOT cells were stained with anti-FOXO3a antibody. FOXO3a-positive immunofluorescence (green) was observed in nuclei of HOZOT cells stained with Hoechst 33258 (blue, arrow) (bars indicate 10 µm).

Next, we undertook a detailed investigation of mRNA and protein expression of FOXO3a in HOZOT and Tconv cells. Three HOZOT and two Tconv cell lines were used for this expression analysis. Results showed that *FOXO3a* mRNA was highly expressed among all HOZOT and Tconv cells (Figure 5A). However, abundant FOXO3a protein expression was detected only in HOZOT cell lines and not in Tconv cells, suggesting the presence of posttranscriptional control of FOXO3a expression in Tconv cells. We also confirmed that FOXO3a protein was localized almost entirely in the nuclei of HOZOTs, indicating that this protein was functionally active (Figure 5B).

### miR-155 decreases FOXO3a expression in JURKAT cells

**Figure 6:**
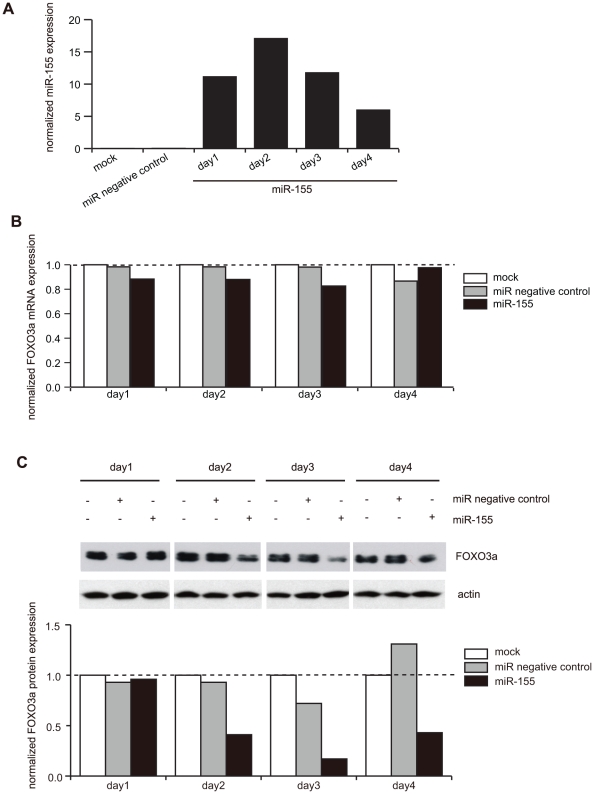
Forced expression of miR-155 downregulates FOXO3a protein expression in JURKAT. *A*. JURKAT cells were transiently transfected with miR-155 precursor. Mature miR-155 levels were monitored by real-time PCR during four day culture period post-transfection. The ratio of miR-155/U6 snRNA in HOZOT-1 was defined as 1.0. miR-155 levels in JURKAT was less than 0.02. *B, C*. Real-time PCR (B) and immunoblot detection (C) of the FOXO3a mRNA and protein expression were performed, respectively, from day one through day four. Densitometric measurement of immunoblot bands is shown after normalizing the data to actin band intensity. Each column in the bar graphs corresponds to each set of the bands right above in immunoblot lanes.

To examine the effect of miR-155 on endogenous FOXO3a protein expression, we utilized JURKAT as host cells because of their high expression of endogenous FOXO3a protein and highly efficient transfectability. First, we examined kinetics of mature miR-155 expression when precursor miR-155 was introduced into JURKAT (over 90% transfection). Since JURKAT expressed endogenous miR-155 at an almost undetectable level, no miR-155 expression was detected in the non-transfected control or the negative miR transfected control. In contrast, JURKAT transfected with miR-155 exhibited a high level of exogenous mature miR-155 expression from day one through day four, reaching its highest level on day two (Figure 6A). With this treatment, repression of FOXO3a protein expression was observed on day two through day four, while its mRNA level remained unchanged (Figure 6B and 6C).

### Gain- and loss-of-function experiments of miR-155 in normal T cells

**Figure 7:**
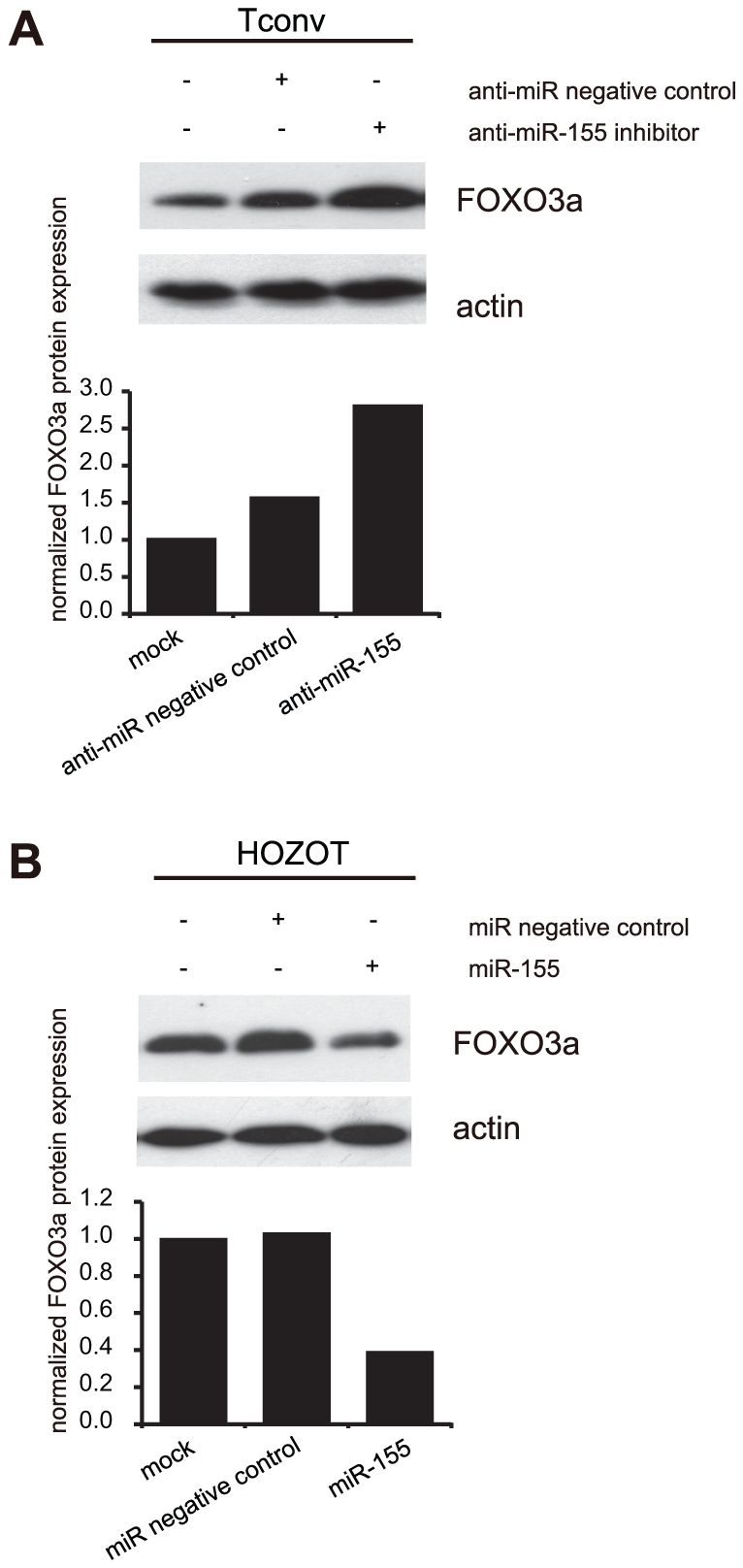
FOXO3a protein expression in normal T cells was also regulated by miR-155. *A.* Tconv cells were transiently transfected with an inhibitor of anti-miR-155. FOXO3a protein expression was detected one day after transfection by immunoblotting using a high sensitivity chemiluminescent system. *B.* HOZOT cells were transiently transfected with the miR-155 precursor. All of the data were representative of more than three independent experiments. Densitometric measurement of immunoblot bands is shown after normalizing the data to actin band intensity. Each column in the bar graphs corresponds to each set of the bands right above in immunoblot lanes.

To confirm the effect of loss or gain of function of miR-155, we introduced either an inhibitor or a precursor of miR-155 in normal T cells including both Tconv cells and HOZOT (over 70% transfection, each). As shown in Figure 7A, transfection with anti-miR-155 increased the FOXO3a protein expression in Tconv probably due to the knockdown effect of anti-miR-155 against endogenous miR-155. The expression of FOXO3a was increased 1.8-fold by anti-miR-155 inhibitor treatment compared with a negative control. On the other hand, transfection with the precursor miR-155 in HOZOT cells decreased the FOXO3a protein expression 2.6-fold compared with a negative control (Figure 7B). Therefore, gain- and loss-of-function experiments support the notion that miR-155 controls the FOXO3a protein expression in normal T cells.

## Discussion

In this study, we demonstrated the unique underexpression of miR-155 in HOZOT cells. Among the known miRNAs, miR-155 is one of the best analyzed, especially in immune competent cells. Its expression has been reported in monocytes [24], macrophages [25] [26], dendritic cells, B cells [27], and T cells [28]. A particularly interesting characteristic of miR-155 is its high inducibility by TLR ligands, TNF-α, or IFNs (monocytes, macrophages, and dendritic cell activation) or antigen receptors (B cell and T cell activations). Therefore, the non-responsive nature of miR-155 in HOZOT stands in sharp contrast to other immuno-competent cells. Interestingly, its unresponsiveness is correlated with the unresponsiveness of HOZOT's FOXP3, the expression of which was not upregulated by TCR stimulation. Since FOXP3 may control BIC/miR155 gene expression, the unresponsiveness of miR155 may be accounted for by the inability to induce FOXP3. However, the mechanisms underlying BIC/miR-155 gene expression have not been elucidated, and further studies are needed to clarify the involvement of FOXP3 in BIC/miR-155 expression. Interestingly, there might be a relationship between underexpression of miR-155 and HOZOT's anergy. As a Treg cell, HOZOT exhibits anergic properties, which is characterized as unresponsiveness to TCR stimulation, i.e., hypoproliferation and low IL-2 production [16]. Although the anergic mechanism of HOZOT is poorly understood, we speculate that suppression of IL-2 gene expression might be controlled by the same or similar molecules which regulate reduced expression of miR-155 and FOXP3.

The physiological functions of miR-155 have been investigated by loss-of–function and gain-of-function studies. It was first reported that miR-155 was expressed at much higher levels in several types of hematopoietic malignancies [29]. Knockout mouse studies have shown that miR-155 plays important roles in immune responses in B cell- and T cell-intrinsic manners based on the observation of impaired germinal center formation [30], inefficient class switching to IgG1 [31], and Th2 bias differentiation of T lineage cells [28]. In innate immunity, miR-155-deficient myeloid dendritic cells have an impaired ability to trigger T cell activation after antigen presentation. Sustained expression of miR-155 had profound effects on hematopoietic populations, resulting in a myeloid proliferative disorder [24]. The target genes controlled by miR-155 are believed to be the following: PU.1, a transcription factor involved in IgG1 switching in B cells [31]; AID, an enzyme mediating class-switch recombination and somatic hypermutation in B-cells [32] [31]; SHIP1, an inositol phosphatase regulating PI3K/Akt pathway in macrophages [26]; and, c-Maf, a potent transactivator of the IL-4 promoter in Th2 differentiating cells [28]. In the context of HOZOT cells, the characteristics of miR-155 in Treg cell biology are most intriguing. First, note that miR-155 controls the properties of Treg, and second, the expression of miR-155 is itself controlled by Treg-specific factors such as FOXP3. For example, miR-155 represses SOCS1 protein expression, resulting in increasing sensitivity to IL-2 and confers on Tregs competitive fitness for proliferation [14]. This is not the case for HOZOT because HOZOT maintains normal sensitivity to IL-2 even with low expression of miR-155.

The significance of low expression of miR-155 was investigated by identifying candidate target genes in HOZOT. There are several examples of target gene analysis where relevant miRNAs are underexpressed in T cells. miR-142-3p regulates the production of cAMP by targeting adenylyl cyclase (AC) 9 mRNA in T cells [15]. However, due to the reduced expression of miR-142-3p, CD4^+^CD25^+^ nTreg cells keep the AC9/cAMP pathway active and exploit a high level of cAMP for their suppressor function. Another example is miR-31 which is markedly underexpressed in human nTreg. miR-31 was shown to directly target FOXP3 by 3′-UTR reporter and anti-miR-31 experiments [33]. In T follicular helper (Tfh) cells, Bcl-6 is a transcriptional repressor which defines Tfh characteristics. Bcl-6 promotes Tfh cell differentiation through the repression of a set of miRNAs which normally prevent effector T cells from expressing Tfh cell signature molecules such as CXCR5, CXCR4, and PD-1. miR-17, miR-18, and miR-20a are involved in CXCR5 upregulation through “repression of the repressor”. In these studies, target genes are intimately relevant to nTreg and Tfh biology [34].

In this study, we identified thirteen mRNA targets that were directly repressed by miR-155 in 3′-UTR reporter assays. Among them, we focused on FOXO3a, a forkhead family gene distinct from FOXP3, the 3′-UTR of which contains four sequences targeted by miR-155. Although FOXO3a mRNAs were expressed at similar levels among all HOZOT and Tconv cells, FOXO3a proteins were expressed in HOZOT cells at higher levels than in Tconv cells, strongly suggesting regulation at translational levels. Direct evidence for the involvement of miR-155 was obtained by introducing miR-155 precursors into JURKAT, in which FOXO3a protein expression was originally at a high level and was reduced by the introduction of miR-155 without changing its mRNA expression level.

FOXO3a has been studied as a transcriptional regulator of cell survival, cell-cycle arrest, stress resistance and tumor suppression [35] [36] [37] [38]. A key feature of FOXO3a is that a variety of post-translational modifications such as phosphorylation, methylation, acetylation and O-linked glycosylation regulate FOXO3a functions, mainly determining nuclear import or export of this protein [39]. Therefore, the fact that FOXO3a is a potent target of miR-155 is intriguing because it provides another layer of control, at the translational level by miRNA, for FOXO3a functions. Recently, there was a report that melanoma cells increased their viability and metastatic activity through miR-182 repression of FOXO3a protein expression, resulting in the reduction of Bim expression [40]. More recently, FOXO3a was identified as a direct target of miR-155. In studies of breast cancer cell lines and tumors, expression of miR-155 and FOXO3a was inversely correlated and FOXO3a protein expression was manipulated by increasing or inhibiting miR-155 expression [41]. In either case, FOXO3a is regarded as a tumor suppressor gene. In HOZOT, the downstream targets of FOXO3a are not known. However, the targets might be different from those involved in cell death or cell cycle arrest since HOZOT maintains proliferative capacity even when FOXO3a is highly expressed and activated. The example of IL-2 withdrawal in T cells [42], in which a FOXO3a downstream target, GILZ, protects T cells from apoptosis, would provide a model of a FOXO3a role in HOZOT.

In conclusion, our study demonstrated a unique example in which miR-155 underexpression, especially non-responsiveness to stimulation, defines a Treg subset, HOZOT. Also, we illustrated a novel relationship between miR-155 and FOXO3a in T cells. Given the significance of both molecules in the immune system, our findings may provide not only an understanding of the multifunctional properties of HOZOT but also further insight into the biology of miR-155 and FOXO3a.

## Materials and Methods

### Ethics Statement

Human umbilical cord blood (UCB) samples were collected from clamped umbilical cords obtained from full-term normal pregnancies with the written consent of the mothers under approval from the Institutional Review Board at the Kurashiki Medical Center. Informed consent was provided according to the Declaration of Helsinki.

### Generation and culture of HOZOTs, nTreg cells, and Tconv cells from umbilical cord blood

All T cells used in this study were generated from mononuclear cells of human UCB as previously reported [16]. Mononuclear cells from the UCB were prepared by gradient centrifugation using Ficoll-Paque (GE Healthcare, Buckinghamshire, UK).

Human regulatory T cell lines, HOZOTs, were generated by co-culture with murine stromal cell lines as previously reported [16]. HOZOT-1, -4, -16, -17 and -18, representative HOZOT cell lines, were used in this study. Briefly, to generate HOZOTs, CD34^−^ mononuclear cells were enriched by negative selection using a MACS CD34^+^ isolation kit (Miltenyi-Biotec, Auburn, CA) and mini-MACS column according to the manufacturer's instructions. The cells were cultured over stromal cells in RPMI 1640 medium supplemented with 10% heat-inactivated FBS, 100 U/ml penicillin, and 50 µg/ml streptomycin at 37°C in 5% CO_2_. HOZOT-1 was established by co-culture with murine stromal cell lines, while MS-5 and HOZOT-4, -16, and -17 were established by co-culture with ST2. Once established as cell lines, HOZOTs were expanded in medium containing 10 ng/ml recombinant human IL-2 (PeproTech, EC, London, UK) by co-culture with stromal cell lines. HOZOTs were purified by Ficoll-Paque to deplete debris from mouse stromal cell lines killed by HOZOTs before experiments.

Normal conventional T (Tconv) cells were obtained by at least one week's cultivation of CD4^+^CD25^−^ cells on plates coated with anti-CD3 mAb (anti-hCD3ε UCHT1, R&D systems, Minneapolis, MN) and anti-CD28 mAb (anti-hCD28, 37407, R&D systems) (CD3/CD28) in the presence of 10 ng/ml IL-2.

nTreg cells, corresponding to naturally occurring Treg cells defined by high FOXP3 expression, were generated by isolating CD25^+^ cells from UCB mononuclear cells as described previously [43]. Briefly, Purified CD25^+^ cells were cultured in wells of plates pre-coated with CD3/CD28 in the presence of 10 ng/ml IL-2. After 24 hours, the cells were transferred to new plates and cultured for two weeks in the presence of 10 ng/ml IL-2.

### miRNA microarray analysis

For the microarray analysis, RNA samples were prepared from two types of cells, HOZOT and Tconv cells generated from the same UCB source. miRNA expression profiling and quantification were performed using mirVanaTM miRNA Bioarray system containing probes for 662 miRNAs in four copies based on miRBase (http://microrna.sanger.ac.uk/) (miRNA Bioarray V2, 1564V1, Ambion, Austin, TX). Samples for microRNA profiling studies were processed by Filgen, Inc. (Filgen, Inc., Japan), according to the company's standard operating procedures. All data is MIAME (Minimum Information About a Microarray Experiment) compliant. The miRNA array data for this paper have been deposited in GEO under accession number GSE22101.

### RNA isolation and quantitative analysis of miR-155

Total RNA was extracted using miRNeasy Mini Kit (QIAGEN, Valencia, CA) according to the manufacturer's instructions. Mature miR-155 was quantified with the TaqMan microRNA assay kit for hsa-miR-155 (Applied Biosystems, Foster City, CA). Ubiquitously expressed U6 small nuclear RNA was used for normalization.

### Flow cytometric analysis of nuclear FOXP3

Intracellular FOXP3 staining was performed using PE-labeled anti-human Foxp3 staining set (eBioScience, San Diego, CA) according to the manufacturer's instructions. Briefly, cells were fixed and permeabilized with Fixation/Permeabilization buffer including paraformaldehyde for 60 min at 4°C, and then stained with PE-conjugated anti-FOXP3 mAb (clone PCH101) or isotype control for 60 min at 4°C. Labeled cells were analyzed by EPICS XL flow cytometer (Beckman Coulter, Miami, FL).

### miR-155 target prediction

Computer-based programs were used to predict potential miR-155 targets. We searched miRBase (http://microrna.sanger.ac.uk/), TargetScan (http://www.targetscan.org/), and miRGen (http://www.diana.pcbi.upenn.edu/cgi-bin/miRGen/v3/Targets.cgi) with default settings.

### Reporter gene construction

Reporter vectors were constructed by introducing a 3′-UTR sequence, which includes the putative miR-155 target site, of each candidate target gene. Briefly, cDNA prepared from HOZOT was used as a template for amplification, and PCR was performed with AccuPrime-*Taq* (Invitrogen, Carlsbad, CA) using the sets of specific primers (Table S1). The amplified fragment of 3′-UTR was first cloned into a pT7Blue TA vector (Merck, Darmstadt, Germany) and then into the luciferase expression vector, psiCHECK-2 (Promega, Madison, WI) through their *XhoI* and *NotI* sites. Site-directed mutagenesis was conducted to mutate predicted binding regions found in FOXO3a 3′-UTR corresponding to the miR-155 seed regions (Table S2).

### Transient transfection and reporter gene assays

The human leukemic T cell line, JURKAT (1×10^5^ cells), was transiently co-transfected with 200 ng of reporter vectors and 5 pmol of Pre-miR miR-155 precursor molecule by Lipofectamin 2000 (Invitrogen) according to the manufacturer's instruction. As a negative control, miR random sequence control (negative control #1; Ambion) was included in the assay. Cell transfectant extracts were prepared 48 hours later and the luciferase activity was measured with the Dual Luciferase Reporter assay kit (Promega) according to the manufacturer's instruction. *Renilla* luciferase activity was normalized to firefly luciferase.

### Modulation of FOXO3a expression by miR-155 in JURKAT cell line

JURKAT was transfected with 5 pmol of Pre-miRTM miR-155 precursor molecule or Pre-miR negative control #1 by Lipofectamin RNAiMAX (Invitrogen) according to the manufacturer's instruction. Total RNA and cell extracts were prepared from day one through day four and FOXO3a expression was measured by qRT-PCR and Western blotting.

### Knockdown or ectopic expression of miR-155 in Tconv or HOZOT cells

miR-155 expression was knocked down in Tconv by transfecting with Anti-miRTM miR-155 Inhibitor (Ambion), whereas miR-155 was ectopically expressed in HOZOT by transfecting with a miR-155 precursor molecule. Transfection was performed with 200 pmol of the inhibitor or the precursor using the GenePulser Xcell electroporation system (Bio-Rad, Hercules, CA). A commercially available control inhibitor (Anti-miRTM miRNA Inhibitors as a negative control #1, Ambion) or precursor (Pre-miRTM negative control #1) was included in the parallel experiment. After transfection, the cells were subjected to further analysis of FOXO3a protein expression.

### Real-time quantitative RT-PCR for FOXO3a mRNA

cDNA was synthesized from the extracted RNA using M-MLV reverse transcriptase (Invitrogen). mRNA levels were quantified by real-time PCR using SYBR Green I Master kit (Roche, Mannheim, Germany) with the LightCycler 480 (Roche). All data were normalized to the 18S rRNA gene, which was measured in the same samples. Primers for FOXO3a were as follows: forward primer 5′- TGCTAAGCAGGCCTCATCTC, reverse primer 5′-GCTCCCTGGACACCCATTCC. Primers for 18S rRNA are as follows: forward primer 5′- GGACACGGACAGGATTGACA, reverse primer 5′- ACCCACGGAATCGAGAAAGA.

### Western blot analysis

Western blotting was performed as described previously [44]. Whole cell extracts were prepared using sodium dodecyl sulfate (SDS) sample buffer solution (125 mM Tris-HCl (pH 6.8), 4.6% SDS, 20% glycerol, and 1.4 M β-mercaptoethanol) at a density of 5×10^7^ cells/ml and 1×10^6^ cells were loaded per lane. Proteins were separated on a 10% SDS-PAGE gel and blotted onto a nylon membrane. After blocking with 10% Block Ace (Dainippon Sumitomo Pharma Co. Ltd., Tokyo, Japan), the membranes were probed with anti-FOXO3a mAb (75D8; Cell Signaling, Danvers, MA) diluted 1:1000 and then with horseradish peroxidase (HRP)-conjugated anti-rabbit IgG (DAKO, Glostrup, Denmark) diluted 1:2000, and finally detected by a chemiluminescent system (Super Signal West Pico, PIERCE, Rockford, IL) or a high sensitivity chemiluminescent system (Immobilon Western Chemiluminescence HRP substrate, MILLIPORE, Bedford, MA). To confirm sample loading and transfer, the membranes were incubated in stripping buffer (100 mM 2-mercaptoethanol, 2% SDS, 62.5 mM Tris–HCl, pH 6.8) for 30 min at 55°C and then re-probed with anti-actin mAb (Chemicon, Temecula, CA) diluted 1:3000 by the same procedure described above. All of the data were representative of more than three independent experiments.

### Immunofluorescent microscopy

For FOXO3a staining, HOZOT cells were washed in PBS and cytospun onto glass slides in a Cytospin 2 (SHANDON Inc., Pittsburgh, PA) at 700 rpm on high acceleration for three min. Cytospins were fixed in 4% paraformaldehyde for 20 min at room temperature. Fixed samples were washed in PBS and blocked in 1% FCS in PBS for 10 min at room temperature. Primary antibody (anti-FOXO3a, prepared in our laboratory) was incubated for 20 min in a humidified chamber at room temperature. The secondary antibody (Alexa 488 goat anti-rabbit IgG, Invitrogen) was incubated for 20 min in a humidified chamber at room temperature. Nuclei were stained with Hoechst 33258. Cells were visualized with a Nikon Optiphot fluorescent microscope (Nikon Inc., Tokyo, Japan).

### Statistics

All data are expressed as the means +/− SD. Statistical differences between groups were analyzed using one-way ANOVA, followed by post hoc Bonferroni/Dunn's tests. Values of p<0.01 were considered to be statistically significant.

## Supporting Information

Table S1 Cloning primer list for 3′-UTR region of target genes.
(DOC)

Table S2 miR-155 predicted binding regions found in FOXO3a 3′-UTR were mutated to disrupt miR155-mediated repression.
(DOC)
