# Supplementary material for: miR-155, a Modulator of FOXO3a Protein Expression, Is Underexpressed and Cannot Be Upregulated by Stimulation of HOZOT, a Line of Multifunctional Treg
Source: PLoS One. 2011 Feb 3;6(2):e16841. doi: 10.1371/journal.pone.0016841 (PMC3033424; doi:10.1371/journal.pone.0016841)
Supplement: Table S1 — Cloning primer list for 3′-UTR region of target genes. (DOC) [file pone.0016841.s001.doc]

**Supplementary Table 1. Cloning primer list for 3’-UTR region of target genes**

| **Target gene** |  | **Sequence** | **Amplicon (bp)** |
| --- | --- | --- | --- |
| BACH1 | sense: | gcactcgagATGTAGTTGTCTCCAGAGCT | 408 |
|  | antisense: | gcagcggccgcCTGTATACTCATAATCCTG |  |
| CDC73 | sense: | gcactcgagGCTTCAAGGAGATGATACC | 990 |
|  | antisense: | gcagcggccgcAATGGTCACTTGATGTACC |  |
| FBXO11 | sense: | gcactcgagAAATATTTGCCCATGCTACAG | 983 |
|  | antisense: | gcagcggccgcACATGCATACACTTTCAGG |  |
| FOS | sense: | gcactcgagGTGCATTACAGAGAGGAGAAA | 728 |
|  | antisense: | gcagcggccgcTGACAATGTCTTGGAACA |  |
| FOXO3a | sense: | gcactcgagTGCGATGGTTTATGGGACGTT | 3134 |
|  | antisense: | gcagcggccgcTTGTCCTGAGTAGTAAGCC |  |
|  | antisense2^*^: | gcagcggccgcTGCTGCACTTGACTTTACC | 2356 |
| HBP1 | sense: | gcactcgagAAGAAGATCAAGGTCTCACCA | 924 |
|  | antisense: | gcagcggccgcACAGCTGTGATTCCTAACC |  |
| IKBKE | sense: | gcactcgagGGGCACATGAGGCATCCTGAA | 703 |
|  | antisense: | gcagcggccgcTGAAGGTGCAGGCCTTGGA |  |
| IL13 | sense: | gcactcgagATTTGCAGAGACAGGACCTGA | 563 |
|  | antisense: | gcagcggccgcACCCCAGTGAGGTAGCAGA |  |
| JARID2 | sense: | gcactcgagTTTGGAGTACTTGCTGTAGGA | 1389 |
|  | antisense: | gcagcggccgcAAGCTCAGTGCAATATGGG |  |
| MYB | sense: | gcactcgagGACATTTCCAGAAAAGCATTA | 1161 |
|  | antisense: | gcagcggccgcTACAAGGCAGTAAGTACAC |  |
| PAPOLA | sense: | gcactcgagGATCGGAAGTCCAGGTTAG | 1715 |
|  | antisense: | gcagcggccgcAAAAGGTCCAAGTTTCCAG |  |
| PICALM | sense: | gcactcgagTAACTCAGTGAAGGCCCATGA | 935 |
|  | antisense: | gcagcggccgcAACATGGCACGAGCCAAAG |  |
| UBQLN1 | sense: | gcactcgagCAGCATTTCTGTATCTTGAAA | 1709 |
|  | antisense: | gcagcggccgcAACTGAGGCATAAAGCAGA |  |

* FOXO3a antisense2 primer is used for mutation experiments.

Sense: gcactcgag Xho I

Antisense: gcagcggccgc Not I
