# Supplementary material for: miR-155, a Modulator of FOXO3a Protein Expression, Is Underexpressed and Cannot Be Upregulated by Stimulation of HOZOT, a Line of Multifunctional Treg
Source: PLoS One. 2011 Feb 3;6(2):e16841. doi: 10.1371/journal.pone.0016841 (PMC3033424; doi:10.1371/journal.pone.0016841)
Supplement: Table S2 — miR-155 predicted binding regions found in FOXO3a 3′-UTR were mutated to disrupt miR155-mediated repression. (DOC) [file pone.0016841.s002.doc]

**Supplementary Table 2.** miR-155 predicted binding regions found in FOXO3a 3’-UTR were mutated to disrupt miR155-mediated repression.

| region | Wt | Mutant |
| --- | --- | --- |
| I | GCATTAA | GGATCCA |
| II | AGCATTAA | AGGATCCA |
| III | AGCATTAA | AGGATCCA |
| IV | AGCATTAA | AGGATCCA |

Mutated nucleotides are shown in red.
